# Headache and comorbidity in children and adolescents

**DOI:** 10.1186/1129-2377-14-79

**Published:** 2013-09-24

**Authors:** Benedetta Bellini, Marco Arruda, Alessandra Cescut, Cosetta Saulle, Antonello Persico, Marco Carotenuto, Michela Gatta, Renata Nacinovich, Fausta Paola Piazza, Cristiano Termine, Elisabetta Tozzi, Franco Lucchese, Vincenzo Guidetti

**Affiliations:** 1 Department of Pediatrics and Child Neuropsychiatry, Sapienza University of Rome, Via Dei Sabelli 108, Rome, Italy; 2 Riberao Preto, Sao Paulo, Brasil; 3 University “Campus Biomedico”, Rome, Italy; 4 II University of Naples, Naples, Italy; 5 Padua University, Padua, Italy; 6 San Gerardo Hospital University of Milano-Bicocca, Monza, Italy; 7 Mondino Institute, Pavia University, Pavia, Italy; 8 Insubria University, Varese, Italy; 9 L’Aquila University, L’Aquila, Italy; 10 Department of Dynamic and Clinical Psychology, Sapienza University of Rome, Rome, Italy

**Keywords:** Headache, Comorbidity, Children, Adolescents

## Abstract

Headache is one of the most common neurological symptom reported in childhood and adolescence, leading to high levels of school absences and being associated with several comorbid conditions, particularly in neurological, psychiatric and cardiovascular systems. Neurological and psychiatric disorders, that are associated with migraine, are mainly depression, anxiety disorders, epilepsy and sleep disorders, ADHD and Tourette syndrome. It also has been shown an association with atopic disease and cardiovascular disease, especially ischemic stroke and patent foramen ovale (PFO).

## Review

### Introduction

Headache is one of the most common somatic complaints in children and adolescents [1]. The prevalence is estimated to be 10–20% in the school-age population, with progressive increase with age, up to values about 27–32% at the age of 13–14 years (considering monthly crisis), 87–94% (considering the presence of headache at least once a year).

Until puberty, it hasn’t been seen gender differences (with a slight male predominance), at a later stage it has been noted an increase among females with a ratio of 2.5:1, except that lasts into adulthood [2,3]. Prevalence of migraine in the pediatric population ranges from 3,3% to 21,4% and it increases from childhood to adolescence [4].

Children and adolescents with headache, and in particular migraine, have worse outcomes, compared to those without migraine, as far as quality of life and school attendance [5] are concerned and they are more likely to have other somatic symptoms (e.g. abdominal pain) [6], anxiety and mood disorders, such as depression [7,8]. Due to its negative impact, on the World Health Organization’s ranking of causes of disability, headache disorders are brought into the 10 most disabling conditions for the two genders, and into the five most disabling for women [9].

In fact, primary headaches, and in particular migraine, is associated with several comorbid conditions.

Comorbidity is the presence of coexisting additional condition in a patient with particular disease index, or the association of non-random two disorders [10].

In children and adolescents, headache and migraine are commonly associated with various diseases, such as psychiatric and neurological comorbidity, in particular depression and anxiety, epilepsy, sleep disorders, ADHD. It also has been shown an association with atopy, cardiovascular disease, especially ischemic stroke and PFO [11-14].

## Headache and psychiatric disorders: anxiety and depression

Since past decades, numerous population- and hospital-based studies have revealed a relationship between migraine or headache and psychopathology in children [15-17]. Depression is more prevalent in headache patients than in the headache-free population [18]. Recently, Pavone et al. (2012) [19] studied the frequency of some comorbidities in primary headaches in childhood.

They enrolled two hundred and eighty children (175 males and 105 females), aged 4 to 14 years, affected by primary headaches. In direct interviews, parents and children gave information about the association of their headaches with different conditions. The Authors found a significant association of primary headache with anxiety and depression.

Migraine is probably the best studied pain disorder in the context of comorbidity with anxiety and/or depression [20]. In a psychiatric setting Masi and collegues [21], in an exploratory study, examine the prevalence of somatic symptoms in a sample of 162 Italian children and adolescents from emotional and/or behavioral disorders. The sample was divided according to gender (96 males, 66 females), age (70 children younger and 92 adolescents older than 12 years), and psychiatric diagnosis (Anxiety, Depression, Depression/Anxiety, Other). The Authors observed that headache was the most frequent somatic symptom in children and adolescents referred for anxiety, depression and behavioral disorders, with a prevalence of females.

Cahill and Cannon [22] defined migraine as a subtype of headache of particular interest for psychiatrists, as they found a linkage between migraine, psychiatric disorders (mainly anxiety and depression), personality traits and stress.

The nature of the relationship between migraine and anxiety is still not clear and we do not know if that relationship is specific to migraine or related to attack frequency [23], even if some evidence confirms that linkage [24]. It is well known that the risk of migraine is higher in patients with comorbid anxiety and/or depression [25] and that anxiety predicts the persistence of migraine and tension-type headache [26]. While only phobic disorder seems to be a predictor of the onset of migraine [27], anxiety, more than depression, predicts long-term migraine persistence, headache-related disability and reduces perceptions of efficacy with acute treatment [26,28]. Phobic disorder is also associated with more frequent and longer migraine attacks, particularly among males [29].

The increased risk of anxiety disorder in children and adolescents with migraine, compared to patients without migraine, is noticed in many studies. Arruda and Bigal [5], in their population-based study, confirmed the higher prevalence of anxious symptoms in children and adolescents with migraine.

In a meta-analysis of 10 studies published between 1996–2011 (406 patients, mean age 11,6 ± 2,3 ys) Ballottin and collegues [23] found that migraine children show more psychological symptoms, detected by using Child Behavior Checklist (CBCL), than healthy controls. They also emphasized the need for studies to compare migraine children with children affected by other chronic pain in order to understand whether the psychopathological profile is migraine- related or chronic pain-related.

Some studies suggest that psychiatric disorders might not specifically relate to migraine, but to chronic illness in general: comparing migraine and chronic non-headache pain samples, Cunningham [30] found no difference in anxiety and depression levels between the two groups with chronic pain, with respect to pain-free controls. Another study [6] comparing headache patients and patients with recurrent abdominal pain did not find differences by the psychological point of view (internalizing vs. externalizing disorders). One of the hypotheses for the comorbidity is that common genetic and/or environmental risk factors may underlie both migraine and psychiatric disorders [27].

Gonda and collegues [31] found that high levels of anxiety and migraine were associated with specific gene polymorphism, supporting the hypothesis of a shared genetic linkage between these two conditions.

Instead other studies show no correlation between migraine, anxiety and depression, as Kowal and Pritchard [32] that studied twenty-three volunteer subjects, compared with 23 (matched) control subjects on self and parental ratings of anxiety, depression, shyness-sensitivity, sleeping difficulties, perfectionism, psychosomatic problems (unrelated to headache), other behavioural disturbances, major life stress events and parental expectations (i.e. achievement orientation). Results indicated that the headache children showed significantly higher shyness-sensitivity, psychosomatic problems and behavioural disturbances and significantly lower parental expectations than the control group children. No other differences were found. While none of the variables were predictive of the frequency or intensity of head pain, measures of anxiety, perfectionism, and life stress events contributed significantly to the prediction of the severity of head pain. Also the study by Laurell et al. [33] show conflicting data. The Authors interviewed 130 schoolchildren and their parents and showed a predominance of comorbidity with other pains rather than psychological and social problems.

In addition to migraine, Chronic daily headache (CDH), defined as 15 or more headaches per month, is associated with increased functional disability and impaired quality of life [34]. Functional disability in children with recurrent headache has also been shown to be a risk factor for psychiatric conditions such as depression [35]. While research in the area of adult headache has made great strides, little is known about the prevalence of psychiatric comorbidity in children with chronic headache conditions. Some researchers have suggested that children with headaches are at increased risk for psychological adjustment problems, including symptoms of anxiety and depression [36,37]. A single published study of a large sample of school-children in Taiwan that did utilize standardized interviews, written by Wang SJ et al. [15], indicated that nearly half (47%) of the sample of 122 children (out of more than 7000 children) who reported chronic headaches had one or more psychiatric disorders, primarily mood or anxiety disorders. Two years later the same Authors identified a higher frequency of suicidal ideation in younger adolescents with migraine with aura or high headache frequency. These associations were independent of depressive symptoms [38]. Parisi P. [39] stressed that antidepressant and antiepileptic usage in adolescents was potentially associated with an increasing suicide risk and that these two medications are frequently used in adolescents with migraine. Moreover, Wang SJ et al. [38] did not exclude the diagnosis of early onset juvenile bipolar disorders (JBD). Although the onset of JBD before the age of 10 is rare and the first manifestation occurs most frequently between the ages of 13 and 15, the diagnosis of JBD is more difficult in children and adolescent populations vs the adult population due to varying symptoms. For example, in children and adolescents, dysphoria is more likely than a euphoric or depressive mood. Asymptomatic intervals rarely exist, yet rapid cycling prevails. In addition, it has been shown that antidepressants in JBD-affected children can have severe adverse effects, particularly the amplification of suicidal ideation. Parisi P., in this respect, indeed, stressed that the possibilities of manic switching and occurrence of suicidal ideation have to be closely monitored when clinicians prescribe antidepressants for treatment of either migraine or depression in adolescents.

Slater et al. (2012) [34] assessed comorbid psychiatric diagnoses in youth with CDH and examined relationships between psychiatric status and CDH symptom severity, as well as headache-related disability. Results showed that 29.6% of CDH patients met criteria for at least one current psychiatric diagnosis. Of those, anxiety disorders were the most common (16.6% of the sample). Mood disorders, on the other hand, were less prevalent (9.5%). The most common anxiety diagnoses were specific phobia (14 of 169), generalized anxiety disorder (10 of 169) and obsessive compulsive disorder (eight of 169). Of the 16 participants with a depressive disorder diagnosis, eight had major depressive disorder, four had a diagnosis of dysthymia, and four met criteria for other mood disorders.

Moreover, 34.9% met criteria for at least one lifetime psychiatric diagnosis. No significant relationship between psychiatric status and headache frequency, duration, or severity was found. However, children with at least one lifetime psychiatric diagnosis had greater functional disability and poorer quality of life than those without a psychiatric diagnosis.

It is, furthermore, important to consider the impact of headache on family life and dynamics.

Children with migraine seem to be characterized by a higher prevalence of familial headache recurrence and parents’ psychiatric disorders than children with other headache subtypes [40]. Only in the case of migraine, higher familial headache recurrence correlates with higher psychiatric comorbidity in children.

The association between migraine and anxiety leads us to think of the need for an integrated, medical and psychological, approach to the taking care of these young patients and their families.

### Headache and sleep disorders

The existence of an intimate relationship between sleep and headache has been recognized for over a century, although the nature of this association is still enigmatic. It is known as sleep deprivation, or, on the contrary, a prolonged sleep, can favor the onset of headache, in particular migraine attack [41]. On the other hand, in many cases, and especially in children, sleep, spontaneous or induced by hypnotics, constitutes the decisive factor for resolution of a migraine attack [42]. Also melatonin seems to effectively reduce the number, intensity and duration of headache attacks per month in the children but the mechanism remains unclear, even though there is much evidence to support the analgesic and anti-inflammatory effects of melatonin [43].

Headaches are known to occur during sleep, after sleep, and in relationship with various sleep stages. Nocturnal migraine attacks can be a result of disrupted sleep, and primary headaches may also emerge during nocturnal sleep, causing sleep disruption [44].

About the variety of phenomena that can disrupt the sleep macrostructure and can impact its restorative function, the periodic limb movements disorder (PLMd) can be considered as the most powerful. No studies are known about the role of PLMd in the pathophysiology of migraine in children. Esposito et al. [45] assess the prevalence of PLMd and migraine and their relationship with disability and pain intensity in a pediatric sample, referred for migraine without aura by pediatricians. This study indicates the potential value of the determination of the PLMd signs, and the importance of the nocturnal polysomnography evaluation in children affected by migraine, particularly when the clinical and pharmacological management tend to fail in the attacks control.

Children who suffer from headache have usually a high rate of sleep difficulties, such as insufficient sleep, co-sleeping with parents, difficulties falling asleep, anxiety related to sleep, restless sleep, night waking, nightmares, and fatigue during the day [46]. Furthermore, an higher prevalence of parasomnias in children, particularly of sleepwalking, bedwetting and pavor, has been documented in migraine patients than in controls [47,48]. The prevalence of sleepwalking in migraineurs seems to swing between 30% and 55% [49]. Different studies propose a model of interaction between headache and sleep [50]. Table 1 shows a model combining clinical data and experimental evidence.

**Table 1:** Models of relations between sleep and headache

**1**	Sleep as trigger factor for headache (excessive, reduced or disrupted, increased deep sleep)
**2**	Sleep as relieving factor for headache
**3**	Sleep disturbance as cause of headache (es. sleep apnea)
**4**	Headache as cause of sleep disturbance (es. attacks occurring during sleep)
**5**	Sleep disorders in headache patients (parasomnias, sleepwalking)
**6**	Sleep related headache:
	a) Temporal relationship (during or after sleep)
	b) Sleep stage relationship:
	1. REM sleep (migraine, cluster headache, chronic paroxysmal hemicrania)
	2. Slow-wave sleep (migraine)
**7**	Headache/sleep association:
	a) Intrinsic origin (modulation through the same neurotransmitters)
	b) Extrinsic origin (i.e. fibromyalgia syndrome)
	c) Reinforcement (bad sleep hygiene)

To date, there are no epidemiological studies to investigate systematically specific comorbidity between headache and the spectrum of sleep disorders. The lack of such studies is compounded by the difficulties of classification of the two disease entities.

Among the epidemiological studies carried out so far, who have analyzed the relationship between sleep and headache, there is a European study, performed by 18,980 telephone interviews. This study showed the presence of chronic headache in the morning in 7.6% of subjects [51]. These, also, than non-sufferers, had more frequently sleep disorders including insomnia, circadian rhythm disorders, snoring, sleep-related breathing disorders, frightening dreams and other dyssomnia.

### Headache and epilepsy

Migraine and epilepsy are the commoner brain diseases and comorbidity of these conditions is well known. This comorbidity is most frequent in childhood and adolescence.

The International Classification of Headache Disorders (ICHD-2) committee recognizes three nosographic entities concerning the relationship between epilepsy and headache: migralepsy, hemicrania epileptica, post-ictal headache [52].

Recent scientific evidences on the ictal epileptic headache have demonstrated that the ‘migralepsy’ concept is exceptional or even it does not exist [53]. On the other hand, migralepsy is neither included in the currently used International League Against Epilepsy (ILAE) seizure classification nor in the recent recommendations of the ILAE Commission on Classification and terminology. In particular, migralepsy, which in the recent ICHD-II is defined as a seizure developing during or within 1 h of a migraine aura, is extremely rare. The concept of migralepsy, according to the current definition, is too narrow and inadequate and it should be revised keeping in the mind that headache or visual symptoms can be the epileptic “aura” of a seizure [54].

Parisi et al. [52] suggest to add, to the forthcoming ICHD-3 classification, the term “ictal epileptic headache” (IEH) which defines a condition diagnosed when a headache attack is the only clinical feature of epileptiform discharges [52-56].

The classification criteria for “ictal epileptic headache” (IEH), was based on twelve well-documented cases that have been published in the literature. The “migraine-epilepsy” sequence, defined, as said, “migralepsy”, may often merely be a seizure starting with an ictal headache, followed by a sensory-motor partial or generalized seizure, which fits into the codified “Hemicrania Epileptica” [53,57].

To date, headache and epilepsy classifications have ignored each other. In the International League Against Epilepsy (ILAE) classification, headache is considered exclusively as a possible semiological ictal phenomenon among the “non-motor” features. In particular, headache is described as a “cephalic”sensation and is not considered as the sole ictal expression of an epileptic seizure. Moreover, headache is not classified as a “pain” (among the “somatosensory” features) or “autonomic” sensation, whereas signs of involvement of the autonomic nervous system, including cardiovascular, gastrointestinal, “vasomotor” and thermoregulatory functions, are classified as “autonomic” features. Now, although still considered a controversial issue, we must consider that headache pain could originate in the terminal nervous fibers (“vasomotor”) in cerebral blood vessels; consequently, headache should be classified as an “autonomic” sensation in the ILAE Glossary and Terminology. Headache could thus be interpreted as the sole expression of an epileptic seizure and classified as an autonomic seizure [53].

In according to these criteria, Parisi et al. [58] propose the term “ictal epileptic headache” for cases in which headache is the sole ictal manifestation, whereas the term “ictal headache” should be applied when the headache, whether brief or long-lasting, is part of a more complex seizure including other sequential or overlapping (sensory-motor, psychiatric or non-autonomic) ictal manifestations.

This new classification proposal (headache as an isolated ictal autonomic manifestation in IEH) has very different prognostic implications because the outcome in people with long-lasting autonomic status epilepticus is very different (i.e., benign) from that of people with additional ictal motor-sensitive semiology. In addition, headache as an autonomic phenomenon is crucial when attempting to understand why headache may be the sole ictal epileptic manifestation: the reasons have been thoroughly explained in Panayiotopoulos syndrome, whereas the threshold required to trigger an ictal autonomic phenomenon is believed to be lower than that required to trigger sensitive sensorial or motor ictal semiology [53].

The criteria of IEH have been proposed by Belcastro et al. [59] to identify the case of headache (as sole ictal manifestation) of epileptic origin in order to promptly obtain an EEG recording and confirm the diagnosis. Table 2, taken from the paper of Parisi et al. [52], shows the proposed criteria for ictal epileptic headache (Diagnostic criteria A–D must all be fulfilled to make a diagnosis of ‘IEH’).

**Table 2:** Proposed criteria for ictal epileptic headache (IEH)

A.	Headache lasting seconds, minutes, hours or days;
B.	Headache that is ipsilateral or contralateral to lateralized ictal epileptiform EEG discharges (if EEG discharges are lateralized);
C.	Evidence of epileptiform (localized, lateralized or generalized) discharges on scalp EEG concomitantly with headache; different types of EEG anomalies may be observed (generalized spike-and-wave or polyspike-and-wave, focal or generalized rhythmic activity or focal subcontinuous spikes or theta activity that may be intermingled with sharp waves) with or without photoparoxysmal response (PPRs)
D.	Headache resolves immediately after i.v. antiepileptic medication

This clinical picture is extremely rare and has only been documented in about 10–12 cases and its epileptic nature is documented with ictal EEG. For this reason, it is difficult to obtain firm conclusions about the frequency of IEH based on epidemiological studies. Using these criteria, we will be able to clarify if IEH represents an underestimated phenomenon or not [59].

Regarding the third entity concerning the relationship between epilepsy and headache, the post-ictal headache, a multicentric italian study from 2006 at 2009 on 142 children, shows that post-ictal headaches were most frequent (62%). Pre-ictal headaches were less common (30%). Inter-ictal headaches were described in 57.6%. Clear migrainous features were present in 93% of pre-ictal and 81.4% of post-ictal headaches. Inter-ictal headaches meet criteria for migraines in 87%. The association between partial epilepsy and migraine without aura is most common and reported in 82% of our patients with peri ictal headache and in 76.5% of patients with post-ictal headache [60]. The term “hemicrania epileptica” should be maintained in the ICHD-II, introduced into the ILAE, and be used to classify all cases in which an “ictal epileptic headache” “coexists” and is associated synchronously or sequentially with other ictal sensory-motor events [55].

As regards the possible causes of comorbidity, the first hypothesis provides a causal relationship of migraine and epilepsy, which seems, however, unlikely considering that some epileptic syndromes such as benign partial epilepsies are observed more frequently [61]. If the association of the two disorders were purely random, the expected prevalence of epilepsy was 1% in migraineurs and the prevalence of migraine was 12% in epileptics, while the literature reports prevalence data significantly higher than expected on basis of random association. The risk for unprovoked seizures was increased in children with migraine with aura and not in patients with migraine without aura [62].

Several epidemiological studies indicate an association of migraine and epilepsy with an increased prevalence of migraine in patients with epilepsy and vice versa. In particular, the prevalence of epilepsy in patients with migraine varies from 1 to 17%, with an average of 5.9%, but this percentage greatly exceeds that of the general population that is approximately 0.5–1%.

The overall prevalence of migraine in children with epilepsy varies from 8 to 15%, with values also increased in children with central-temporal EEG spikes (63%) and epilepsy with absences (33%) [63,64]. The risk of migraine is more than twice as high in subjects with epilepsy both in probands than in relatives, compared to people without epilepsy [65].

As a second hypothesis has been suggested a causal unidirectional relationship, for instance in case of migraine can cause cerebral ischemia or cerebral damage, and consequently epilepsy, or in the case of "migralepsy", where migraine aura can trigger a seizure. More often a seizure triggers an attack of headache post-critical, often with migraine characteristics, in this case it has been hypothesized that epilepsy can trigger migraines through activation of the trigeminal-vascular system or through mechanisms-encephalic trunk [66].

However, the unidirectional hypothesis has been contradicted in a study for verified the relationship between migraine and epilepsy in 395 adult seizure patients, conducted by Marks et al. [67], since in the majority of patients with migraine and epilepsy (66/79, 84%) attacks were completely independent. A third hypothesis requires that common environmental risk factors, such as head injury, can cause both migraine and epilepsy. In fact, it has been found an increased risk of migraine in people with epilepsy caused by head trauma, and that in each subgroup of epilepsy, defined on the basis of seizure type, age at onset, etiology, and family history [68]. On the other hand, the presence of shared environmental factors do not explain the increased risk of migraine in patients with idiopathic epilepsy and several studies have documented the association between migraine and rolandic epilepsy and idiopathic occipital epilepsy [69,70].

Headache may occur before, during or after an epileptic seizure, as well as vomiting. In idiopathic occipital epilepsy, crises are, in fact, often characterized by vomiting associated with visual symptoms, focal seizures and headache.

The existence of a possible constitutional common ground between migraine and epilepsy was initially proposed on the basis of significantly greater familiarity of migraine in epileptics (28%) and for epilepsy in migraineurs (2–3%) [70].

The genetic hypothesis (fourth hypothesis) was tested by Ottman et al. [65], which have suggested a higher incidence of migraine in families with genetic forms of migraine than those with non-genetic forms and that the relatives of patients with migraine and epilepsy had an increased incidence of epilepsy compared to the relatives of patients with only epilepsy. However, this hypothesis was not confirmed by other studies [68].

Subsequent work reported data in favor of possible genetic factors common to the two conditions. In fact, in some families with idiopathic temporal lobe epilepsy was found a higher prevalence of migraine [71,72]. An extended family with several individuals with occipital and temporal lobe epilepsy was also featured, which segregated with an autosomal dominant mode of transmission; epileptic patients had migraines with aura are independent of seizures [73].

Most obvious is the association between migraine with aura (MWA) and epilepsy. In fact, in a study of 134 children and adolescents with headache, there was a high prevalence of MWA (30.4%) than other types of primary headache in children with seizures [74]. Another study of population-based case–control study documented that the risk of seizures was increased in children with MWA and not in those with migraine without aura (MOA) [75]. In addition, in a study of adult patients, the frequency of MWA was significantly higher in patients with epilepsy in comorbidity (41%) compared to patients only with migraine (25.8%) [76].

Finally, considering the comorbidities as a result of an alteration in brain excitability, Leninger et al. [76] investigated whether the clinical features associated with diffuse cortical depression (the so-called “Spreading Depression,” CSD) were more severe in patients with comorbidities. Despite the frequency of epileptic seizures and syndromes did not differ between patients with epilepsy alone, compared to subjects with comorbidy, migraine with aura, worsening pain with physical activity, phonophobia and photophobia were significantly more frequent in patients with comorbidities compared with patients with epilepsy or migraine alone.

These differences are in favor of the hypothesis that the link between migraine and epilepsy is based on the CSD as an expression of neuronal hyperexcitability. The altered neuronal excitability may cause an increased sensitivity to the CSD resulting in an increased activation of the trigeminal nociceptive fibers and consequently in more severe migraine attacks [76].

Therefore it is likely that the altered neuronal excitability threshold, involved in migraine and epilepsy, and due to altered levels of neurotransmitters, is attributable to genetic factors, in particular the disorders of membrane ion channels, the so-called channelopathies.

The epilepsy and migraine, in fact, share common pathogenetic mechanisms partially related to the dysfunction of ion channels, it is assumed, therefore, that channelopathies may be the link between epilepsy and migraine, particularly when these disturbances are in comorbidy.

However, when headache and epilepsy overlap as a result of the crossing of the cascade of events at the cortical level, in both of the events (CSD and epileptic focus), their onset and propagation are triggered when these events reach a certain threshold, which is lower for CSD than for seizure. These two phenomena may be triggered by more than one pathway converging (at cortical level) upon the same destination: depolarization and hypersynchronization [53].

Finally, further studies are warranted to better delineate the complex link between epilepsy and migraine.

### Headache and general medical conditions

Compared to comorbidity between headache and general medical conditions, an interesting epidemiological study, led by Lateef et al. [77] on the child population, highlights the correlation between headache and other general medical conditions, including asthma, hay fever and frequent ear infections. The 41.6% of children with headache had at least one of these conditions, and in general, the group examined had a probability of 3.2 times higher to present two of the above conditions and a probability of 13.6 times greater to submit all three. The increased comorbidity between headache and general medical conditions was found from 4 to 11 years.

Other conditions most frequently observed in children with frequent or severe headaches are: ADHD, especially as regards hyperactive/impulsive behavior [78], learning disabilities, stuttering, anemia, obesity, bowel disease. Regarding the girls, it was found that most of those who had frequent headaches had their first menstrual cycle before the age of 12 years [77].

It was also found a higher comorbidity of headache, in particular migraine, with atopic disorders (asthma, rhinitis or eczema), studied in a sample of children presenting with such disorders. The prevalence of migraine was significantly higher in children with atopic disorders than those without. In particular, the greater association was detected with rhinitis [12].

Recent researches suggest that obesity was significantly correlated with migraine frequency and disability in children, as well as in adult population studies. Translational and basic science research shows multiple areas of overlap between migraine pathophysiology and the central and peripheral pathways regulating feeding. Specifically, neurotransmittors such as serotonin, peptides such as orexin, and adipocytokines such as adiponectin and leptin have been suggested to have roles in both feeding and migraine. A relationship between migraine and body mass index exists, and therefore, interventions to modify body mass index may provide a useful treatment model for investigating whether modest weight loss reduces headache frequency and severity in obese migraineurs [79].

The effect of obesity and weight change on headache outcomes may have important implications for clinical care.

Recently, Verrotti et al. [80] investigated the real impact of a weight loss treatment on headache in a sample of obese adolescents. In all, 135 migraineurs, aged 14–18 years, with body mass index (BMI) greater than or equal 97th percentile, participating in a 12-month-long program, were studied before and after treatment. The program included dietary education, specific physical training, and behavioral treatment.

Decreases in weight, BMI, waist circumference, headache frequency and intensity, use of acute medications, and disability were observed at the end of the first 6-month period and were maintained through the second 6 months. Both lower baseline BMI and excess change in BMI were significantly associated with better migraine outcomes 12 months after the intervention program.

So, initial body weight and amount of weight loss may be useful for clinicians to predict migraine outcomes [80].

### Headache and cerebro and cardio-vascular diseases

Although migraine is an accepted cause of cerebral infarction in adults, this association is not recognized in children. The mean annual incidence of stroke in children is about 2.5 per 100,000 [78].

The causes of cerebral infarction in children may include: heart disease, vascular disease, blood disorders, primary hypercoagulable states or congenital metabolic disorders, but 50% of strokes are considered idiopathic [81].

In the adult population is generally accepted that cerebral infarction may occur during a migraine attack [82]. In young adults, ischemic strokes could be the result of migraine in a percentage ranging from 10% to 27% [83]. In contrast, in children, the diagnosis of stroke caused by migraine is still questioned, in fact, until now, only a few cases have been reported in subjects under the age of 16 years [84,85]. In most patients, the ischemic stroke occurred in a middle cerebral artery territory [85] but may be involved also areas of the brain sprayed from basic.

In particular, there appears to be a complex relationship in a bidirectional association between migraine and stroke, including migraine as a risk factor for cerebral ischemia, migraine caused by cerebral ischemia, migraine as a cause of stroke, the presence of a common cause for migraine and cerebral ischemia or migraine associated with subclinical vascular injury of the brain.

Some studies of young adults seem to confirm this association [86,87]. A history of migraine with aura seems to be more common among victims of ischemic stroke than among controls and an acute attack of migraine may precede, accompany or follow a thromboembolic transient ischemic attack or a stroke, this seems to occur more often among migraineurs compared patients without migraine [88,89]. Adults suffering from migraine with aura are at increased risk of cardiovascular disease and stroke [90], but it is necessary to consider that in adults, the analysis of this association is complicated by a frequent presence of additional risk factors such as smoking, hypertension and diabetes mellitus. In children, these and other potential confounding factors are much less common. There are relationships arising from small clinical samples of pediatric age who demonstrate the association of migraine with dyslipidemia [91], hyperhomocysteinemia and genetic variants related to homocysteine which appear to be risk factors for the development of stroke in children [92].

Furthermore, in a national representative sample of children, the severe or recurrent headache was associated with higher levels of adiposity measured by the body mass index (BMI) [77].

In general, below the age of 55, migraine with aura is a risk factor for ischemic strokes. However, it’s important to point out that part of the latter, can be linked to the presence of a patent foramen ovale (PFO).

The PFO is the result of incomplete fusion of the septum “primum” and “secundum”, which normally occurs shortly after birth, when the left atrial pressure exceeds that of the right atrium.

Epidemiological studies have shown a clear comorbidity between migraine with aura and PFO. In fact, the available data suggest that PFO is more common in women with migraine with aura (present in about 50% of cases) and that migraine with aura is more common in patients with PFO [93-95]. The mechanism underlying the possible relationship between migraine and PFO is not yet very clear: is there a causal relationship with migraine attacks, or have common genetic factors? The pathophysiological mechanism is considered a passage of microemboli and vasoactive chemicals through the PFO, which would circumvent the filtering pulmonary triggering migraine symptoms. The widespread cortical depression, which is the mechanism behind the migraine aura, could be favored by the presence of a PFO. Among the various hypotheses, it seems interesting to Pierangeli et al., who claim that a particular genetic predisposition could lead to a co-development of atrial septal abnormalities and migraine [96]. In fact, if the aura has occurred due to a malfunction of the cerebral perfusion, the symptoms should occur with a sudden onset and not gradual. It’s likely that the association of migraine and PFO is random, given the frequency of both disorders.

Recently, Steenblik et al. [97] sought to examine the familial risk of isolated interatrial shunt, caused by either atrial septal defect or patent foramen ovale, and explore associated comorbidities of stroke, transient ischemic attack (TIA), and migraine using a population database.

They found that there is a strong familial inheritance pattern for isolated interatrial shunt, with significantly higher risk of interatrial shunt among affected patients’ siblings, first-, and second-degree relatives. Relatives of affected individuals also had a higher risk of TIA, a trend toward an increased risk for stroke, but no increased risk of migraine headache.

The relevance of genetic factors with respect to the preparation and transmission of PFO and migraine with aura is still under discussion [98-100].

### Headache and tourette syndrome

Tourette syndrome (TS) is recognized as one of the most common childhood movement disorders, characterized by motor and phonic tics often associated with neurobehavioral comorbidities, such as obsessive-compulsive disorder. Neurotransmitter dysregulation, particularly involving the serotonin system, has been implicated in the pathogenesis of TS, obsessive-compulsive disorder, and migraine headache. The frequency of migraine headache in a clinic sample of TS subjects was nearly 4-fold more than the frequency of migraines reported in general population. In particular, of 100 patients with TS, 25 (25.0%) satisfied the diagnostic criteria for migraine headache, significantly greater than the estimated 10% to 13% in general adult population and the estimated 2% to 10% in general pediatric population [101].

The first study that has examined the comorbidity between Tourette syndrome and headache was conducted by Barabas et al. (1984) [102]. The authors studied the incidence of migraine among children with Tourette’s Syndrome (TS). Among 60 children with TS (mean age of 11.9 yrs), migraine was prevalent in 26.6%. This figure is substantially greater than that reported for general population of school-aged children (4.0–7.4%) or for 2 control groups consisting of 72 children with seizure disorders and 62 children with learning disabilities. The prevalence rates for these two control groups were 11.3% and 8.0%, respectively.

Subsequently, in 1986, Lacey D.J. [103] have shown a correlation between Tourette’s syndrome and several other disorders in children, including: thought and behavioral disorders, sleep disturbances, headaches, and school difficulties-including attention deficit disorder.

A recent study of Ghosh et al. (2012) [104] analyzed the frequency of occurrence of headaches in children and adolescents with TS to address their possible inclusion as a comorbidity.

Using a prospective questionnaire, administered directly, the author interviewed a total sample size of 109 patients with TS ≤21 years of age. The questionnaires were then analyzed according to the International Headache Society’s diagnostic criteria. The author found that headaches were present in 55% of patients, with two most common headache types being migraine headaches and tension-type headaches. The rate of migraine headache within the TS group was found to be 4 times greater than that of general pediatric population, as reported in literature. In addition, the rate of tension-type headache was found to be more than 5 times greater than that of general pediatric population. Overall, the high rates of migraine and tension-type headache within this population support the proposition that headaches are a comorbidity of TS.

### Headache and ADHD

Primary headache syndromes (eg, migraine and tension-type headache [TTH]) and attention-deficit/hyperactivity disorder (ADHD) are prevalent in childhood and may cause impairment in social and academic functioning.

In particular, Migraine and ADHD are highly prevalent, affecting between 5 to 10% of the pediatric population [4,105]. Coincidentally, the burden caused by both neuropsychiatric disorders reaches a common range of negative outcomes impairing quality of life [106,107], school achievement [108,109], social [7,110], and family functioning [111,112]. Thus, studying the association of both conditions is of utmost clinical importance.

According to a systematic review of clinical studies on psychological functioning and psychiatric comorbidity of migraine in children, there is no evidence that ADHD is more frequently diagnosed in this group compared with no headache controls [7].

In a cross-sectional epidemiological study specifically designed to examine this association, we have found that migraine are not comorbid to ADHD overall, but are comorbid to hyperactive-impulsive behavior [88]. In this study ADHD was assessed according to the fourth edition of the *Diagnostic and Statistical Manual of Mental Disorders* (DSM-IV) criteria by the validated Brazilian version of the Multimodal Treatment Study of Children with ADHD – Swanson, Nolan, and Pelham IV (MTA-SNAP-IV) scale [113] fulfilled by parents and teacher. Mental health status was assessed with the validated Brazilian version of the Child Behavior Checklist (CBCL) [114]. The prevalence of ADHD was not significantly different comparing children with migraine to controls (no headache). For inattention symptoms, no significant differences were found. The prevalence of hyperactivity-impulsivity symptoms was 8.1% in children without headache, 23.7% in children with migraine (relative risk [RR] = 2.6; 95% confidence interval [CI] = 1.6–4.2), and 18.4% in children with probable migraine (RR = 2.1; 95% CI = 1.4–3.2). According to the multivariate analyses, ADHD or inattention symptoms were not predicted by headache subtypes or headache frequency. On the other hand, hyperactivity-impulsivity symptoms were significantly associated with any headache (p < 0.01), tension-type headache (TTH) (p < 0.01), or migraine (p < 0.001) [88].

An association between childhood migraine and inattention symptoms have been reported by some populational [115,116] and clinical [6] studies. However, the findings must be understood in the context of some methodological limitations. The behavior rating scales adopted by these studies add symptoms of inattention and hyperactivity/impulsivity in the same domain preventing the distinction between them. Among the 11 questions comprising the attention domain in the CBCL, only three capture symptoms of inattention (*“Can’t concentrate, can’t pay attention for long”, “Daydreams or gets lost in his/her thoughts”*, and *“Stares blankly“*). The remaining questions focus on hyperactivity, impulsivity, executive dysfunctions and lack of coordination (*“Acts too young for his/her age”, “Can´t sit still, restless, or hyperactive”, “Confused or seems to be in a fog”, “Impulsive or acts without thinking”, “Nervous, high strung, or tense”, “Nervous movements or twitching”, “Poor school work”,* and *“Poorly coordinated or clumsy”*) [117]. Likewise, of the five questions that encompass the hyperactivity scale of the Strengths and Difficulties Questionnaire (SDQ), two are destined to identify inattention symptoms and three to hyperactivity/impulsivity [118]. Adopting the MTA-SNAP-IV scale we could separate both dimensions of ADHD symptoms in our study [88].

In accordance to our findings, neuropsychological studies with clinical samples have found no attentional impairment in children with migraine compared to controls, in spite of a rather impulsive response profile [119-121].

Given the possible comorbidity between migraine and hyperactivity-impulsivity symptoms, providers and educators should be aware of the association.

## Conclusions

Primary Headaches In Childhood and Adolescence are often associated with,and deeply influnced by,many comorbid situations.

In this review are analyzed the most relevant of them.

It is foundamental to take care of any kind of comorbidity to establish the most effective treatment strategy.

## Competing interest

The authors declare that they have no competing interest.

## Authors’ contributions

All authors contributed to the writing of each paragraph of the manuscript. All authors read and approved the final manuscript.
